# Extracts or Active Components from Acorus gramineus Aiton for Cognitive Function Impairment: Preclinical Evidence and Possible Mechanisms

**DOI:** 10.1155/2020/6752876

**Published:** 2020-08-25

**Authors:** Yan Li, Xi-Le Zhang, Yan-Ran Huang, Yan-Yan Zheng, Guo-Qing Zheng, Li-Ping Zhang

**Affiliations:** ^1^Department of Neurology, Zhejiang Hospital, Hangzhou, Zhejiang 310013, China; ^2^Department of Neurology, The Second Affiliated Hospital and Yuying Children's Hospital of Wenzhou Medical University, Wenzhou, China; ^3^The First Affiliated Hospital of Zhejiang Chinese Medical University, China

## Abstract

Extracts or active components from Acorus gramineus Aiton (EAAGA) have been clinically used for cognition impairment more than hundreds of years and are still used in modern times in China and elsewhere worldwide. Previous studies reported that EAAGA improves cognition impairment in animal models. Here, we conducted a preclinical systematic review to assess the current evidence of EAAGA for cognition impairment. We searched 7 databases up until June 2019. Methodological quality for each included studies was accessed according to the CAMARADES 10-item checklist. The primary outcome measures were neurobehavioral function scores evaluated by the Morris water maze test, electrical Y-maze test, step-down test, radial eight-arm maze test, and step-through test. The secondary outcome measures were mechanisms of EAAGA for cognition function. Finally, 34 studies involving 1431 animals were identified. The quality score of studies range from 1 to 6, and the median was 3.32. Compared with controls, the results of the meta-analysis indicated EAAGA exerted a significant effect in decreasing the escape latency and error times and in increasing the length of time spent in the platform quadrant and the number of platform crossings representing learning ability and memory function (all *P* < 0.01). The possible mechanisms of EAAGA are largely through anti-inflammatory, antioxidant, antiapoptosis activities, inhibition of neurotoxicity, regulating synaptic plasticity, protecting cerebrovascular, stimulating cholinergic system, and suppressing astrocyte activation. In conclusion, EAAGA exert potential neuroprotective effects in experimental cognition impairment, and EAAGA could be a candidate for cognition impairment treatment and further clinical trials.

## 1. Introduction

With the average life expectancy increasing, there is concern about the proportion of cognitive impairment in the global population, which results from degeneration of the brain and very high prevalence in elderly individuals [1]. The World Health Organization estimates that the number of people over the age of 60 will be around 2 billion in 2050, while the number of cognitive impairment patients is expected to rise rapidly along with the aging population worldwide [2, 3]. However, so far, clinical trials have not identified efficacious neuroprotective therapies for cognitive impairment patients [4]. Thus, given the huge translational gap between the animal studies and clinical trials, seeking or developing innovative neuroprotectants is urgently needed.

For more than a millennium, traditional Chinese medicine (TCM), a main form of complementary and alternative medicine, has been used in Asian countries, especially in China, Japan, and Korea, to alleviate various symptoms of cognitive deficits and to facilitate learning and memory [5]. Acorus gramineus Aiton (AGA) (record 2322 (http://www.theplantlist.org.)), the dry rhizomes of Acorus gramineus Solander (Shi Changpu), is listed officially in the Chinese Pharmacopoeia and used in oriental medicines for more than hundreds of years to treat neurological disorders. AGA possessed various pharmacological effects on the central nervous system, including neuroprotective effects [6, 7], central inhibitory effects [8], inhibitory effects on excitotoxic neuronal death [9], and stroke [10], and amelioration in learning and memory [5]. AGA may be effective for the improvement of amnesia [9]. AGA contains different extract fractions: volatile oil, composing mainly of *β*-asarone (63.2–81.2%), and *α*-asarone (8.8–13.7%) [11], as well as water extract, ethyl ether extract, ethyl acetate extract, N-butanol extract, and the defatted decoction fractions. AGA is often used as a component in some Chinese herbal formulas. Among 75 of the most famous Chinese herbal formulas characterized as improving intelligence both in ancient and modern time in China, more than half contain AGA, such as Kai-Xin-San [12] and Chong-MyungTang [13].

Systematic reviews are believed to be preferred; only data that from systematic reviews will be considered as the highest level of medical evidence basis for the levels of evidence from the Centre of Evidence-Based Medicine in Oxford [14]. Preclinical systematic reviews are a powerful approach to analyze and synthesize the results of an intervention from animal data into a useful document that can help to shape further basic research, optimize the experimental studies, and enhance the success rate of future clinical trials [15]. Thus, we conducted a preclinical systematic review to assess the current evidence of extracts or active components from Acorus gramineus Aiton (EAAGA) and active component for animal models of cognitive impairment.

## 2. Materials and Methods

### 2.1. Search Strategies

Experimental studies of EAAGA for cognitive impairment were identified in the databases, including PubMed, Embase, Web of Science, Wanfang database, China National Knowledge Infrastructure (CNKI), CBM, and VIP information database. All searches were performed from inception to June 2019. Studies about assessing the effectiveness of AAGA for improving cognitive function impairment in animals were identified. The search terms were as follows: (Acorus tatarinowii Schott OR Rhizoma acori graminei OR Acorus calamus OR Acorus gramineus Soland OR acorus gramineus aiton OR Acori graminei rhizoma OR Acori tatarinowii rhizoma OR grassleaf sweetfalg Rhizome) AND (cognitive function impairment OR amnesia OR dementia OR Alzheimer's disease).

### 2.2. Inclusion Criteria

Experimental studies on EAAGA for cognitive impairment models were included, regardless of publication status or animal species, gender, age, and methods of model establishment. The primary outcome measurements were Morris water maze test (MWM test), electric Y-maze test (EY-M test), radial eight-arm maze test (RAM test), Step down test (SD test), and/or Step through test (ST test). The secondary outcome measures were mechanisms of EAAGA for learning and/or memory function.

### 2.3. Exclusion Criteria

Exclusion criteria were prespecified as follows: (1) the article was a review, case report, comment, clinical trial, abstract, or editorial; (2) the article was a clinical or *in vitro* study; (3) the article was not a research about cognitive impairment model; (4) EAAGA was used as combination; (5) there was no control group; and (6) the article was a duplicate publication.

### 2.4. Data Extraction

The information of each included study was extracted: (1) author and publication year, animal model species, method of anesthesia, and random method; (2) characteristics of animals, including species, sex, animal number, and weight; (3) treatment information from treatment and control groups, including drug, dose, method of treatment, timing for initial treatment, frequency, and duration of treatment; and (4) outcome measures, sample size, and corresponding data including mean value, standard deviation, and intergroup differences. If outcomes were presented at different time points, we extracted data from the last time point. If studies utilized dose gradient of the drug, we extracted data from the highest dose of EAAGA and active component since the dose-response relationship. If the data were incomplete or presented in graphs, we tried to contact the authors for data needed or calculated using relevant software. Information of the mechanism studies of EAAGA and active component for cognitive impairment models among the included articles was extracted.

### 2.5. Quality Assessment

The methodological quality of included studies was evaluated by two independent reviewers using Collaborative Approach to Meta-Analysis and Review of Animal Data from Experimental Studies (CAMARADES) 10-item checklist [16]. For calculating an aggregate quality score, each item of this scale was attributed one point.

### 2.6. Statistical Analysis

Meta-analysis was conducted via RevMan version 5.3. To estimate the effect of EAAGA on cognitive impairment, the random effects model and standard mean difference (SMD) with 95% confidence intervals (CIs) were calculated. Heterogeneity was assessed via *I*^2^ statistics test. If probability value was less than 0.05, the difference was considered statistically significant. In addition, to explore potential sources of high heterogeneity, subgroup analyses were performed according to animal species and models. Difference between groups was determined by partitioning heterogeneity and utilizing the *χ*^2^ distribution with degrees of freedom (df).

## 3. Results

### 3.1. Study Selection

**Figure 1:**
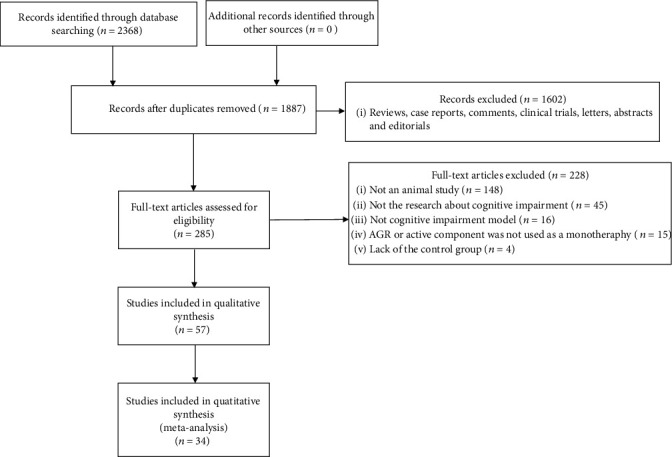
Flow diagram.

We identified 2368 potentially relevant papers after systematical search from six databases. After removing duplicates, 1887 studies remained. By reading titles and abstracts, 1602 articles were excluded that were reviews, case reports, comments, abstracts, clinical trials, letters, or editorials. After reading the remaining 285 full-text articles, 228 studies were excluded for at least one of following reasons: (1) not an animal study; (2) the article was not a research about cognitive impairment; (3) the study did not access the effects of AGA or active component on the animal model of cognitive impairment; (4) EAAGA was not used as a monotherapy; and (5) lack of control group. Ultimately, 34 eligible articles [5, 6, 10, 11, 17–46] were selected **(**Figure 1**)**.

### 3.2. Characteristics of Included Studies

Sixteen studies [5, 6, 10, 11, 17–27, 37] were published in English, and 18 studies were in Chinese between 1999 and 2019. In total, 34 studies with 1431 animals were included. Ten species were referred, including Sprague-Dawley (SD) rat (*n* = 236, 16.49%), Wistar rats (*n* = 130, 9.08%), Kunming mice (*n* = 530, 37.04%), ICR mice (*n* = 236, 16.49%), NIH mice (*n* = 168, 11.74%), A*β*PP/PS1 double-transgenic mice (*n* = 26, 1.82%); APPswe/PS1dE9 double transgenic mice (*n* = 22, 1.54%), C57BL/6 mice (*n* = 24, 1.68%), senescence-accelerated prone-8 (SAMP8) mice (*n* = 26, 1.82%), and FMR1gene knock mice (*n* = 33, 2.31%). The weight of SD rats ranged from 200 g to 650 g, the weight of Wistar rats used ranged from 30 g to 250 g, and the weight of mice ranged from 17 g to 50 g. Twenty-two studies used male rodents, 1 study used female rodents, 5 study used both female and male rodents, and the remaining 6 studies did not provide gender details. Sodium pentobarbital was used to induce anesthesia in 8 studies, and chloral hydrate was used in 2 studies [20, 21], 1 study [41] used phenytoin sodium, 1 study [17] used CO_2_, and 1 study [10] used isoflurane, while the remaining 21 studies did not report the type of anesthetics. Cognitive impairment models were induced by lead [17], noise stress [18], LPS [19], amyloid beta 1-42 [11, 21, 26, 28, 29, 37, 41, 46], D-gal plus AlCl_3_ [22], scopolamine [5, 24, 30, 34–36, 42, 45], ethanol [5, 32, 34–36], sodium nitrite [5, 32], corticosterone [23], Ibotenic acid [25], chronic restraint stress [31], pentobarbital sodium [32], D-galactose [33, 38], AlCl_3 [_40_]_, streptozotocin (STZ) [43], pent ylenetet razol (PTZ) [44], and NaNO_2_ [34–36]. As an intervention, fourteen studies [6, 17, 20, 22, 23, 26, 27, 32, 35, 37, 39, 41, 42, 46] used *β*-asarone, eight studies [18, 19, 21, 24, 33, 38, 40, 44] used *α*-asarone, three studies [10, 25, 44] utilized AGA, twelve studies [5, 11, 22, 28–32, 35, 36, 43, 45] used essential oil, seven studies [11, 28, 29, 33–36] researched water extract, four studies [11, 28, 29, 32] used defatted decoction, and one study [18] researched ethyl acetate extract. Normal distilled water control was used in 2 studies [17, 33]; Tween 80 control was used in 6 studies [5, 6, 18, 20, 27, 32]; normal saline control was used in 24 studies; 0.5% methylcellulose solution containing 1% Tween 80 control was used in 1 study [24], and 2% propylene glycol containing 2% polyethylene glycol stearate control was used in 1 study [43]. Neurobehavioral function indices as primary outcome measures were carried out by the Morris water maze test (MWM test) (*n* = 28), step-down test (SD test) (*n* = 6), electrical Y-maze test (EY-M test) (*n* = 3), step-through test (ST test) (*n* = 4), and radial eight-arm maze test (RAM test) (*n* = 3). The characteristics of the 34 studies are shown in Table 1.

### 3.3. Study Quality

The quality scores of the 34 included studies varied from 1/10 to 6/10 with the average of 3.32. One study [40] got 1 point; 11 studies [29, 31–36, 38, 39, 42, 44] got 2 points; 9 studies [5, 6, 18, 24, 25, 27, 30, 43, 45] got 3 points; 4 studies got 4 points; 7 studies got 5 points; and 2 studies [20, 37] got 6 points. Thirty-four studies were published. Sixteen studies described control of temperature [6, 10, 17–26, 30, 37, 41, 45]. Random allocation was declared in 28 studies [5, 6, 11, 17, 19–23, 26–28, 30–39, 41–46]; 1 study [42] used random block allocation method, and 2 studies used the method of random digit table [34, 41]. Two studies [23, 37] described the use of blinded assessment of outcome. Thirteen studies did not use anesthetics with significant intrinsic neuroprotective activity, and the remaining 21 studies did not report the type of anesthetics [5, 6, 18, 19, 24, 27, 30–40, 42–45]. Sixteen studies reported compliance with animal welfare regulations [5, 10, 11, 17–22, 24, 27, 28, 37, 41, 43, 45]. Four studies mentioned statement of potential conflict of interests [11, 20, 28, 37]. None of the included studies reported allocation concealment, sample size calculation, and the utilization of animal or model with relevant comorbidities. The quality scores for the included studies are shown in Table 2.

### 3.4. Effectiveness

**Figure 2:**
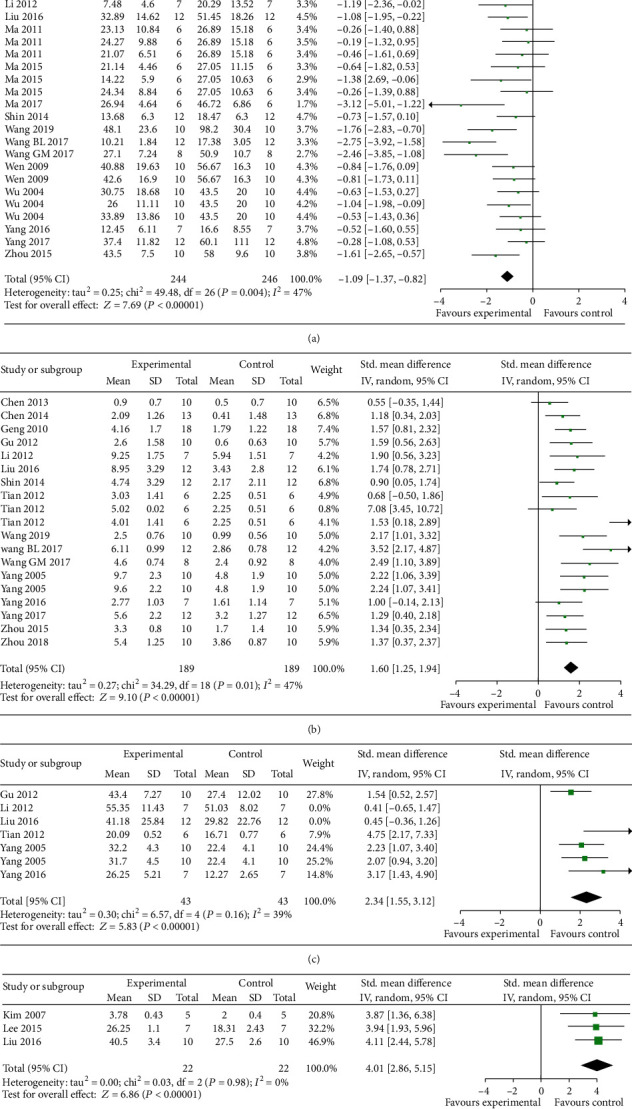
The forest plot in Morris water maze test. Effects of EAAGA for decreasing the escape latency (a) in spatial test, increasing crossing numbers (b), increasing exact time (c), and increasing percentage of time (d) in platform quadrant in probe test compared with control group.

The Morris water maze test, including the probe test and the spatial test, was conducted in 28 studies [6, 10, 11, 17, 19, 20, 22, 23, 25–31, 33–39, 41–46]. Twenty-seven studies reported the spatial test using the escape latency as an outcome measure. Meta-analysis of 20 studies with 27 comparisons showed EAAGA significantly decreased the escape latency compared with the control (*n* = 490, SMD = −1.09, 95% CI [−1.37 to −0.82], *P* < 0.00001; heterogeneity: *χ*^2^ = 49.48, df = 26 (*P* = 0.004); *I*^2^ = 47%; Figure 2(a)). In the probe test, meta-analysis of 16 studies [17, 19, 20, 22, 26, 27, 29–31, 33, 34, 37, 38, 41, 44, 45] with 19 comparisons showed EAAGA were significant for increasing number of platform crossings (*n* = 398, SMD = 1.60, 95% CI [1.25 to 1.94], *P* < 0.00001; heterogeneity: *χ*^2^ = 34.29, df = 18 (*P* = 0.01); *I*^2^ = 47%; Figure 2(b)) compared with controls. Meta-analysis of 6 studies [17, 20, 22, 29, 34, 44] with 7 comparisons showed a significant effect of EAAGA in increasing the length of time spent in platform quadrant compared with control (*n* = 144, SMD = 1.78, 95% CI [0.90 to 2.67], *P* < 0.0001; heterogeneity: *χ*^2^ = 22.41, df = 6 (*P* = 0.001); *I*^2^ = 73%). As the values of *I*^2^ were greater than 50%, we sequentially omitting each study; two studies [20, 22] were removed and markedly reduced the heterogeneity (*n* = 86, SMD = 2.34, 95% CI [1.55 to 3.12], *P* < 0.00001; heterogeneity: *χ*^2^ = 6.57, df = 4 (*P* = 0.16); *I*^2^ = 39%; Figure 2(c)). Two studies [20, 22] used relatively large doses of *β*-asarone that might have potential toxic effects [47]. Meta-analysis of 3 studies [20, 23, 25] for increasing percentage of time in the platform quadrant (*n* = 44, SMD = 4.01, 95% CI [2.86 to 5.15], *P* < 0.00001; heterogeneity: *χ*^2^ = 0.03, df = 2 (*P* = 0.98); *I*^2^ = 0%; Figure 2(d)). Three studies [17, 22, 23] showed there were not a significant difference in improving the swimming velocity compared with controls.

**Figure 3:**
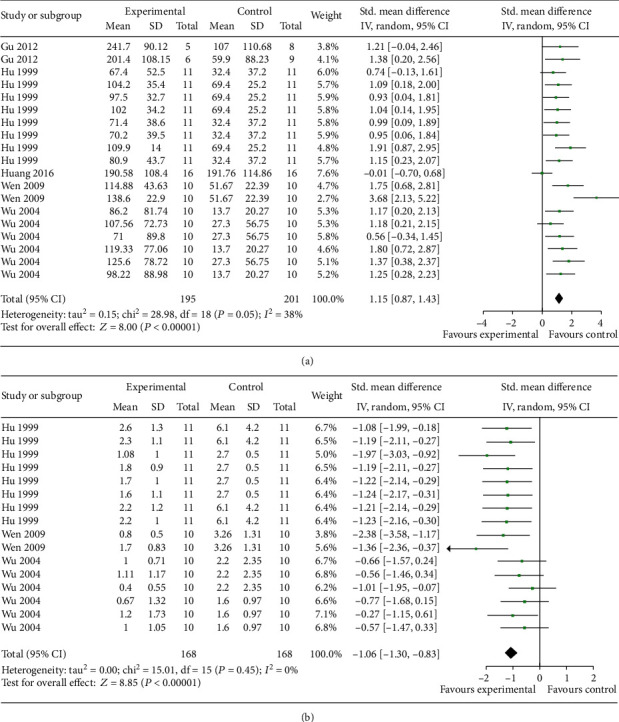
The forest plot in Step-down test. Effects of EAAGA for increasing right reaction latency in the retention test (a) and decreasing the error times in the retention test (b) compared with control group.

The step-down test, including the training test which represents learning ability and retention test which represents memory ability, was conducted in 6 studies [5, 32, 34–36, 40]. Meta-analysis of 5 studies with 19 comparisons showed EAAGA were significant for increasing right reaction latency in the retention test (*n* = 396, SMD = 1.15, 95% CI [0.87 to 1.43], *P* < 0.00001; heterogeneity: *χ*^2^ = 28.98, df = 18 (*P* = 0.05); *I*^2^ = 38%; Figure 3(a)) and 1 study [5] for increasing right reaction latency (*P* < 0.05) in the training test. Meta-analysis of 3 studies [32, 35, 36] with 16 comparisons showed EAAGA were significant for decreasing the error times (*n* = 336, SMD = −1.06, 95% CI [−1.30 to −0.83], *P* < 0.00001; heterogeneity: *χ*^2^ = 15.01, df = 15 (*P* = 0.45); *I*^2^ = 0%; Figure 3(b)) in the retention test and 1 study [5] for decreasing the error times (*P* < 0.05) in the training test.

**Figure 4:**
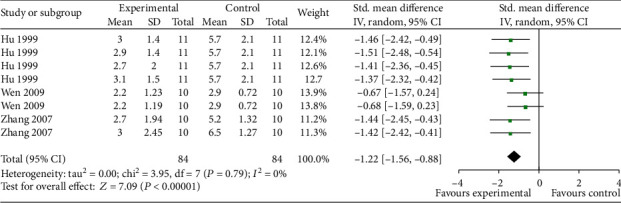
The forest plot in Electrical Y-maze test. Effects of EAAGA for decreasing error reaction times compared with control group.

The electrical Y-maze test was conducted in 3 studies [5, 32, 36]. Meta-analysis of 3 studies showed EAAGA were significant for decreasing error reaction times (*n* = 168, SMD = −1.22, 95% CI [−1.56 to −0.88], *P* < 0.00001; heterogeneity: *χ*^2^ = 3.95, df = 7 (*P* = 0.79); *I*^2^ = 0%; Figure 4).

**Figure 5:**
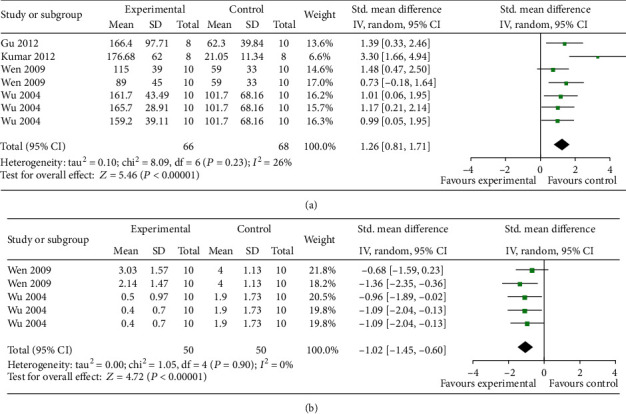
The forest plot in Step-through test. Effects of EAAGA for decreasing latency in the retention test (a) and decreasing the number of errors in the retention test (b) compared with control group.

The step-through test was conducted in 4 studies [24, 34–36]. Meta-analysis of 4 studies with 7 comparisons showed EAAGA were significant for decreasing latency in the retention test (n = 134, SMD = 1.26, 95% CI [0.81 to 1.71], P < 0.00001; heterogeneity: *χ*^2^ = 8.09, df =6 (P = 0.23); *I*^2^ = 26%; Figure 5(a)) and 2 studies [35, 36] with 5 comparisons showed EAAGA significantly decreased the number of errors in the retention test (*n* = 100, SMD = −1.02, 95% CI [−1.45 to −0.60], *P* < 0.00001; heterogeneity: *χ*^2^ = 1.05, df = 4 (*P* = 0.90); *I*^2^ = 0%; Figure 5(b)) compared with controls.

**Figure 6:**
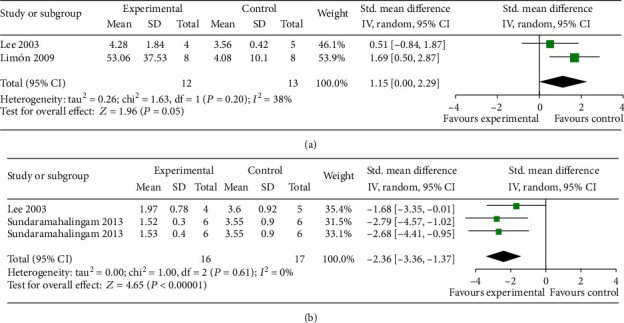
The forest plot in Eight-arm maze test. Effects of EAAGA for increasing correct choices (a) and decreasing the number of errors (b) compared with control group.

The eight-arm maze test was conducted in 3 studies [10, 18, 21]. Meta-analysis of 2 studies [10, 21] showed EAAGA were significant for increasing number of correct choices (*n* = 25, SMD = 1.15, 95% CI [0.00 to 2.29], *P* = 0.05; heterogeneity: *χ*^2^ = 1.63, df = 1 (*P* = 0.20); *I*^2^ = 38%; Figure 6(a)) and 2 studies [10, 18] with 3comparisons showed EAAGA significantly decreased the number of errors in the training test (*n* = 33, SMD = −2.36, 95% CI [−3.36 to −1.37], *P* < 0.00001; heterogeneity: *χ*^2^ = 1.00, df = 2 (*P* = 0.61); *I*^2^ = 0%; Figure 6(b)) compared with controls.

### 3.5. Neuroprotective Mechanisms

**Figure 7:**
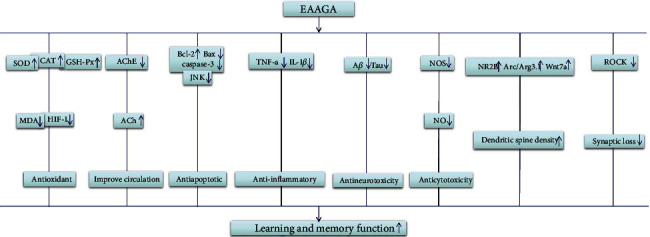
A schematic representation of possible mechanisms of EAAGA for improving learning and memory function. The possible mechanisms of different active ingredients are as follows: (1) AGA: the dry rhizomes of Acorus gramineus Solander can inhibit apoptosis and stimulate cholinergic system. (2) Essential oil: AGA contains up to 4.86% essential oil, which displayed antioxidation effects by decreasing the levels of MDA and increasing the levels of SOD, exhibited anticytotoxicity effects via decreasing NOS activity, exerted antineurotoxicity effects by decreasing A*β* plaques depositions, and improved cognitive function by decreasing the activity of AChE. (3) *β*-Asarone: a major component of essential oil (63.2–81.2%) displayed antioxidation effects by decreasing the levels of MDA and HIF, increasing the levels of SOD, CAT, and GSH-Px; exerted antiapoptotic activity through regulating CaMKII/CREB/Bcl-2 signaling pathway and decreasing the levels of Bax mRNAs, caspase-3 mRNA, and JNK; inhibited synaptic loss through reducing ROCK expression; mediated synaptogenesis via Arc/Arg3.1 and Wnt pathway; improved circulation by decreasing the activity of AChE; and exerted antineurotoxicity by decreasing A*β* plaques depositions. (4) *α*-Asarone: another major component of essential oil (8.8–13.7%) exerted antioxidation effects by increasing CAT, SOD, and GSH-Px.; displayed anti-inflammatory activity through reducing the expression of proinflammatory mediators; improved circulation via decreasing the activity of AChE; and exerted antineurotoxicity by decreasing A*β* plaques depositions. (5) Water extract: displayed antioxidation effects by decreasing the levels of MDA and increasing the levels of SOD, exerted antineurotoxicity by decreasing A*β* plaques depositions; and improved cognitive function by decreasing the activity of AChE. (6) Defatted decoction: exerted antineurotoxicity by decreasing A*β* plaques depositions and displayed anticytotoxicity effects via decreasing the activity of NOS.

The mechanisms of neuroprotection of EAAGA on cognitive impairment were studied in 34 included articles [5, 6, 10, 11, 17–46] as follows: (1) reduction of oxidative reactions by increasing the activity of SOD [30, 35, 39, 41, 43] activity, while decreasing the activity of SOD and AChE [18, 24], decreasing the levels of MDA [24, 30, 33] and nitric oxide [21], decreasing the mRNA levels of hsp 70, increasing the levels of VC, VE, and GSH, and increasing the activity of CAT and G6PD [18]; (2) inhibition of apoptosis by increasing the mRNA levels of Bcl-2, BDNF, CREB [6, 23, 42], Bcl-w and Bcl-2 [26], and c-jun [35], decreasing the mRNA levels of Bax [23], increasing the expression of BDNF, CREB [23], Bcl-w, and Bcl-2 [26], decreasing the expression of caspase-3, p-JNK [26], and BACE1 [19], and preventing cell loss [10], A*β*, and Tau protein [38]; (3) repression of inflammatory reactions by decreasing the expression of TNF-*α* and IL-1*β* mRNA levels [19]; (4) repression of autophagy by decreasing LC3, ROCK, and beclin1 expression and increasing p62, GAP43, MAP2, and SYN expression [27]; (5) protection of cerebrovascular by increasing rCBF and the Na-K-ATP activity, decreasing pyruvic acid contents, and decreasing the mRNA levels of ET-1, eNOS, and APP [22]; (6) promotion of cognitive function by increasing the levels of 5-HT, NE, DA, and NE [5] and suppression of astrocyte activation [37]; (7) stimulation of cholinergic system by increasing AchE and ChAT neurons [25]; (8) improvement of memory impairments through regulation of synaptogenesis, which is mediated via Arc/Arg3.1 and Wnt pathway [17]; (9) neuroprotection through damage of Akt pathway [40]; (10) inhibition of neurotoxicity by decreasing the expression of DCx and nestin, decreasing nestin positive cells [11], decreasing A*β* plaques depositions, and decreasing NOS activity [29]; (11) regulation of synaptic plasticity by increasing the expression of SYP and GluR1 [20, 46] and decreasing the expression of GAP-43 and PSD-95 [46]; and (12) inhibition of chronic stress by decreasing plasma cortisol levels [41]. Characteristics of mechanism studies of EAAGA on experimental ischemic stroke are shown in Table 3 and Figure 7.

## 4. Discussion

As far as we know, it is the first preclinical systematic review that determined the efficacy of EAAGA for learning and memory function. In the present study, 34 studies with 1431 animals showed that EAAGA significantly improve learning and memory function, suggesting the potential neuroprotective functions of EAAGA in cognitive function impairment. However, given methodological weaknesses, the overall available evidence from the present study should be interpreted cautiously.

Some limitations should be considered while interpreting this study. First, we only searched databases in Chinese and English. The absence of studies published in other languages may cause certain degree selective bias [48]. Second, the methodological quality of included studies showed some inherent drawback. Most of the research had methodological flaws in aspects of blinding, randomization, allocation concealment, sample size calculation, and lacking statement of potential conflict of interests [49, 50]. The studies without adequate sample sizes, allocation concealment, or randomization may result in inflated estimates of treatment efficacy [51, 52]. Lower quality trials could attribute to statistically significant 30–50% exaggeration of treatment efficacy [53]. Third, no study adopted animals with comorbidities, which would have created more relevant models for human pathology [49]. Thereby, the results should be interpreted cautiously.

The poor design of animal research hindered the translation of animal research into effective preclinical drug treatments for human disease [54, 55]. Thus, it is necessary to take a rigor experimental design to overcome methodology quality issues for further research. The Animal Research: Reporting of In Vivo Experiments (ARRIVE) [56, 57] is a reporting guideline consisting of a 20-item checklist that provides recommendations on Introduction, Methods, Results, and Discussion which were recommended to be utilized as guidelines when designing and reporting animal research on EAAGA for improving the cognitive function impediment. Meanwhile, many drugs that exerted significant effects in animal researches failed to translate into effective clinical drug treatments [58, 59]. One of the possible reasons is the application of drug doses and the timing of drug administration in animal models that are inapplicable for human disease [55]. In the present study, doses of EAAGA and timing for initial administration in animal models were inconsistent among the 34 included studies. Thus, we suggest further studies to determinate the optimal gradient doses and timing of administration in animal models of cognition impairment.

The present study showed that EAAGA had cognitive enhancing effects through different mechanisms as follows: (1) reduction of oxidative reactions by increasing the activity of SOD [30, 35, 39, 41, 43] activity, while decreasing the activity of SOD and AChE [18, 24], decreasing the levels of MDA [24, 30, 33] and nitric oxide [21], decreasing the mRNA levels of hsp 70, increasing the levels of VC, VE and GSH, and increasing the activity of CAT and G6PD [18]; (2) inhibition of apoptosis by increasing the mRNA levels of Bcl-2, BDNF, CREB [6, 23, 42], Bcl-w and Bcl-2 [26], and c-jun [35], decreasing the mRNA levels of Bax [23], increasing the expression of BDNF, CREB [23], Bcl-w, and Bcl-2 [26], decreasing the expression of caspase-3, p-JNK [26], and BACE1 [19], and preventing cell loss [10], A*β*, and Tau protein [38]; (3) repression of inflammatory reactions by decreasing the expression of TNF-*α* and IL-1*β* mRNA levels [19]; (4) repression of autophagy by decreasing LC3, ROCK, and beclin1 expression and increasing p62, GAP43, MAP2, and SYN expression [27]; (5) protection of cerebrovascular by increasing rCBF and the Na-K-ATP activity, decreasing pyruvic acid contents, and decreasing the mRNA levels of ET-1, eNOS, and APP [22]; (6) promotion of cognitive function by increasing the levels of 5-HT, NE, DA, and NE [5] and suppression of astrocyte activation [37]; (7) stimulation of cholinergic system by increasing AchE and ChAT neurons [25]; (8) improvement of memory impairments through regulation of synaptogenesis, which is mediated via Arc/Arg3.1 and Wnt pathway [17]; (9) neuroprotection through damage of Akt pathway [40]; (10) inhibition of neurotoxicity by decreasing the expression of DCx and nestin, decreasing nestin positive cells [11], and decreasing A*β* plaques depositions, decreased NOS activity [29]; (11) regulation of synaptic plasticity by increasing the expression of SYP and GluR1 [20, 46] and decreasing the expression of GAP-43 and PSD-95 [46]; and (12) inhibition of chronic stress by decreasing plasma cortisol levels [41]. However, cellular and molecular alteration mechanisms of EAAGA and active components for cognition impairment have not been clearly explored yet, which presented an exciting investigative direction of further studies. All 5 measuring methods for learning and memory ability were used in the 34 included studies, which showed that the measuring methods for cognition impairment were inconsistent. The diverse measuring methods for learning and memory ability need further study.

## 5. Conclusions

Although some factors such as study quality may undermine the validity, EAAGA exert potential neuroprotective effects in cognition impairment. In addition, AGA and active components may be a promising candidate for clinical trials.

## Figures and Tables

**Table 1:** Basic characteristics of the included studies.

Study (years)	Species (sex, *n* = experimental/control group)	Weight	Random method	Model (method)	Anesthetic	Method of administration		Outcome index (time)	Intergroup differences
						Experimental group	Control group		
Yang et al. [17]	SD rats (mix, 7/7)	NR	NR	Chronic lead-induced dysmnesia model	CO2	*β*-Asarone (2.5, 10, and 40 mg kg^−1^, ip); from 9 to 11 weeks old; once daily for 3 weeks	Distilled water (same volume, ip); from 9 to 11 weeks old; once daily for 3 weeks	(1) MWM test (escape latency)	(1) *P* < 0.001
								(2) MWM test (swimming speed)	(2) *P* > 0.05
								(3) MWM test (time spent in target quadrant)	(3) *P* < 0.05
								(4) MWM test (times crossed the platform)	(4) *P* > 0.05
								(5) Dendritic spine density	(5) *P* < 0.001

Wei et al., 2013	A*β*PP/PS1 double-transgenic mice (13/13)	NR	NR	A*β*PP/PS1 double-transgenic mice	NR	*β*-Asarone (7 and 21 mg kg^−1^, ig); onset the experiment; once daily for 4 months	Tween 80 (same volume, ig); onset the experiment; once daily for 4 months	(1) MWM test (escape latency)	(1) *P* < 0.001
								(2) Cell viability	(2) *P* < 0.05

Sundaramahalingam et al. [18]	Wister strain albion rats (male, 6/6)	200-220 g	NR	Noise stress induced memory impairment model	NR	*α*-Asarone (9 mg kg^−1^, ip); onset the experiment; once daily for 30 d	Tween 80 (same volume, ip); onset the experiment; once daily for 30 d	(1) RAM test (number of errors)	(1) *P* < 0.05
								(2) Hsp 70 mRNA levels	(2) *P* < 0.05
								(3) Ache activity	(3) *P* < 0.05
								(4) SOD/CAT/GPx activity	(4) *P* < 0.05
								(5) VC/VE/GSH levels	(5) *P* < 0.05
								(6) G6PD activity	(6) *P* < 0.05
	Wister strain albion rats (male, 6/6)	200-220 g	NR	Noise stress exposed rats	NR	Ethyl acetate extract (50 mg kg^−1^, ip); onset the experiment; once daily for 30 d	Tween 80 (same volume, ip); onset the experiment; once daily for 30 d	(1) RAM test (number of errors)	(1) *P* < 0.05
								(2) Hsp 70 mRNA levels	(2) *P* < 0.05
								(3) Ache activity	(3) *P* < 0.05
								(4) SOD/CAT/GPx activity	(4) *P* < 0.05
								(5) VC/VE/GSH levels	(5) *P* < 0.05
								(6) G6PD activity	(6) *P* < 0.05

Shin et al. [19]	C57BL/6 mice (male, 12/12)	25-28 g	NR	LPS-induced cognitive handicap model	NR	*α*-Asarone (7.5, 15, and 30 mg kg^−1^, ig); 3 days before the LPS injection; once daily for 3 d	Normal saline (same volume, ig); 3 days before the LPS injection; once daily for 3 d	(1) MWM test (escape latency)	(1) *P* < 0.05
								(2) MWM test (times crossed the platform)	(2) *P* < 0.05
								(3) TNF-*α*/IL-1*β* mRNA levels	(3) *P* < 0.05
								(4) CA1 neurons count	(4) *P* < 0.05
								(5) TUNEL-labeled cells count	(5) *P* < 0.05
								(6) BACE1/Iba1 protein expressions	(6) *P* < 0.05

Ma et al. [11]	Six-week-old NIH mice (male, 6/6)	20-25 g	NR	A*β*_1-42_-induced AD model	Sodium pentobarbital	Water extract (20 mg g^−1^, ig); after the first MWM test; once daily for 3 weeks	Normal saline; (same volume, ig); after the first MWM test; once daily for 3 weeks	(1) MWM test (escape latency)	(1) *P* < 0.05
								(2) A*β* positive cells count	(2) *P* < 0.05
								(3) DCx expression	(3) *P* < 0.05
								(4) Nestin positive cells count	(4) *P* < 0.05
	Six-week-old NIH mice (male, 6/6)	20-25 g	NR	A*β*_1-42_-induced AD model	Sodium pentobarbital	Essential oil (20 mg g^−1^, ig); after the first MWM test; once daily for 3 weeks	Normal saline; (same volume, ig); after the first MWM test; once daily for 3 weeks	(1) MWM test (escape latency)	(1) *P* < 0.05
								(2) A*β* positive cells count	(2) *P* < 0.05
								(3) DCx expression	(3) *P* < 0.05
								(4) Nestin positive cells count	(4) *P* < 0.05
	Six-week-old NIH mice (male, 6/6)	20-25 g	NR	A*β*_1-42_-induced AD model	Sodium pentobarbital	Defatted decoction (20 mg g^−1^, ig); after the first MWM test; once daily for 3 weeks	Normal saline; (same volume, ig); after the first MWM test; once daily for 3 weeks	(1) MWM test (escape latency)	(1) *P* < 0.05
								(2) A*β* positive cells count	(2) *P* < 0.05
								(3) DCx expression	(3) *P* < 0.05
								(4) Nestin positive cells count	(4) *P* < 0.05

Liu et al. [20]	APPswe/PS1dE9 double transgenic mice (male, 11/11)	NR	NR	APPswe/PS1dE9 double transgenic mice	Chloral hydrate	*β*-Asarone (21.2, 42.4, and 84.8 mg kg^−1^, ig); onset the experiment; once daily for 2.5 months	Tween 80 (same volume, ig); onset the experiment; once daily for 2.5 months	(1) MWM test (escape latency)	(1) *P* < 0.05
								(2) MWM test (time spent in target quadrant)	(2) *P* < 0.05
								(3) MWM test (times crossed the platform)	(3) *P* < 0.05
								(4) SYP/GluR1 expression	(4) *P* < 0.05

Limón et al. [21]	Wistar rats (male, 8/8)	230–250 g	NR	A*β*_1-42_-induced AD model	Chloral hydrate	*α*-Asarone (10 mg kg^−1^, i.h.); after injection of amyloid-*β*; once daily for 16 d	Normal saline; (same volume, ig); after injection of amyloid-*β*; once daily for 16 d	(1) RAM test (percentage of correct responses)	(1) *P* < 0.001
								(2) Nitrite levels	(2) *P* < 0.05

Li et al. [22]	Wistar rats (female, 7/7)	150–180 g	NR	D-gal and AlCl_3_ induced AD model	Sodium pentobarbital	*β*-Asarone (25, 50 and 100 mg kg^−1^, i.h.); 28 d after injection of AlCl3 and D-gal; once daily for 14 d	Normal saline; (same volume, i.h); after the first MWM test; once daily for 14 d	(1) MWM test (escape latency)	(1) *P* < 0.05
								(2) MWM test (time spent in target quadrant)	(2) *P* < 0.05
								(3) MWM test (times crossed the platform)	(3) *P* < 0.05
								(4) MWM test (swimming speed)	(4) *P* > 0.05
								(5) ET-1, eNOS, and APP expression	(5) *P* < 0.05
								(6) Lactic acid and pyruvic acid content	(6) *P* < 0.05
								(7) Na^+^- K^+^ ATPase activity	(7) *P* < 0.05
								(8) rCBF	(8) *P* < 0.05

Zhang et al. [5]	Aged Kunming mice (male, 10/10)	40-50 g	NR	Aged mice	NR	Essential oil (0.02, 0.04, and 0.08 g kg^−1^, orally); onset the experiment; once daily for 15 d	Tween 80 (same volume, orally); onset the experiment; once daily for 15 d	(1) SD test (escape latency)	(1) *P* < 0.01
								(2) SD test (number of errors)	(2) *P* < 0.05
	Aged Kunming mice (male, 10/10)	40-50 g	NR	Scopolamine-induced dysmnesia model	NR	Essential oil (0.02, 0.04, and 0.08 g kg^−1^, orally); onset the experiment; once daily for 15 d	Tween 80 (same volume, orally); onset the experiment; once daily for 15 d	(1) SD test (escape latency)	(1) *P* < 0.05
								(2) SD test (number of errors)	(2) *P* < 0.01
	Aged Kunming mice (male, 10/10)	40-50 g	NR	Ethanol-induced dysmnesia model	NR	Essential oil (0.02, 0.04, and 0.08 g kg^−1^, orally); onset the experiment; once daily for 15 d	Tween 80 (same volume, orally); onset the experiment; once daily for 15 d	(1) SD test (escape latency)	(1) *P* < 0.01
								(2) SD test (number of errors)	(2) *P* < 0.01
	Aged SD rats (male, 10/10)	550-650 g	NR	Aged rats	NR	Essential oil (0.02, 0.04, and 0.08 g kg^−1^, orally); onset the experiment; once daily for 30 d	Tween 80 (same volume, orally); onset the experiment; once daily for 30 d	(1) EY-M test (number of errors)	(1) *P* < 0.01
								(2) NE, DA and 5-HT level	(2) *P* < 0.01
								(3) AChE activity	(3) *P* < 0.01
									(4) *P* < 0.01
	Aged SD rats (male, 10/10)	550-650 g	NR	Sodium nitrite-induced dysmnesia model	NR	Essential oil (0.02, 0.04, and 0.08 g kg^−1^, orally); onset the experiment; once daily for 30 d	Tween 80 (same volume, orally); onset the experiment; once daily for 30 d	(1) EY-M test (number of errors)	(1) *P* < 0.05
									(2) *P* < 0.01

Lee et al. [10]	SD rats (male, 5/7)	250-280 g	NR	MCAO-induced cognitive impairments model	Isoflurane	AGA (100 mg kg^−1^, po); after occlusion; once daily for 21 d	Normal saline; (same volume, i.h); after occlusion; once daily for 21 d	(1) MWM test (escape latency)	(1) *P* < 0.05
								(2) Cell density	(2) *P* < 0.05

Lee et al. [23]	SD rats (male, 7/7)	200-220 g	NR	Chronic corticosterone-exposed model	Sodium pentobarbital	*β*-Asarone (50, 100, and 200 mg kg^−1^, ip); 30 min prior to the CORT; once daily for 21 d	Normal saline; (same volume, ip); 30 min prior to the CORT; once daily for 21 d	(1) MWM test (swimming speed)	(1) *P* > 0.05
								(2) serum CORT levels	(2) *P* < 0.05
								(3) BDNF and CREB expression	(3) *P* < 0.05
								(4) Bax and Bcl-2 mRNAs expression	(4) *P* < 0.05

Kumar et al. 2012	ICR mice (8/8)	NR	NR	Scopolamine-induced amnesic model mode	NR	*α*-Asarone (3, 10, and 30 mg kg^−1^, po); 15 d before scopolamine injection; once daily for 15 d	0.5% methylcellulose solution containing 1% Tween 80 (same volume, po); 15 d before scopolamine injection; once daily for 15 d	(1) SD test (escape latency)	(1) *P* < 0.01
								(2) AchE activity	(2) *P* < 0.001
								(3) MDA levels	(3) *P* < 0.001
								(4) SOD activity	(4) *P* < 0.05

Kim et al. [25]	SD rats (male, 5/5)	260–280 g	NR	Ibotenic acid-induced amnesia	Sodium pentobarbital	AGA (100 mg kg^−1^,ip); after surgery; once daily for 3 weeks	Saline (same volume, ip); after surgery; once daily for 3 weeks	(1) MWM test (escape latency)	(1) *P* < 0.001
								(2) ChAT positive neurons count	(2) *P* > 0.05
								(3) AchE neurons density	(3) *P* < 0.05

Geng et al. [26]	SD rats (male, 20/20)	220-240 g	NR	A*β*_1-42_-induced AD model	Sodium pentobarbital	*β*-Asarone (12.5, 25, or 50 mg kg^−1^, ig); 3 d after A*β* (1-42) hippocampus injection; once daily for 28 d	Saline (same volume, ip); 3 d after A*β* (1-42) hippocampus injection; once daily for 28 d	(1) MWM test (escape latency)	(1) *P* < 0.05
								(2) MWM test (times crossed the platform)	(2) *P* < 0.05
								(3) Annexin V-positive cells	(3) *P* < 0.05
								(4) Caspase-3 and Caspase-3 mRNA express	(4) *P* < 0.05
								(5) Bcl-2 and Bcl-2 mRNA levels	(5) *P* < 0.05
								(6) Bcl-w, and Bcl-w mRNA express	(6) *P* < 0.05
								(7) P-JNK express	(7) *P* < 0.05

Chen et al. [27]	SAMP8 mice (13/13)	NR	NR	SAMP8 mice	NR	*β*-Asarone (34 mg kg^−1^, ig); onset the experiment; once daily for 2 months	Tween 80 (same volume, ig); onset the experiment; once daily for 2 months	(1) MWM test (number of platform crossing)	(1) *P* < 0.05
								(2) MWM test (escape latency)	(2) *P* < 0.05
								(3) LC3-positive cells	(3) *P* < 0.05
								(4) Beclin express	(4) *P* < 0.05
								(5) 1p62 express	(5) *P* < 0.05
								(6) ROCK1 express	(6) *P* < 0.05
								(7) GAP43, MAP2 and SYN expression	(7) *P* < 0.05
								(8) GAP43, MAP2 and SYN positive cells	(8) *P* < 0.05
								(9) Lipofuscin-positive cells	(9) *P* < 0.05

Ma et al. [28]	NIH mice (male, 6/6)	18-20 g	NR	A*β*_1-42_-induced AD model	Sodium pentobarbital	Water extract (0.02 g g^−1^, ig); after the first MWM test; once daily for 3 weeks	Normal saline (same volume, ig) after the first MWM test; once daily for 3 weeks	(1) MWM test (escape latency)	(1) *P* < 0.05
								(2) Beta-amyloid IOD	(2) *P* < 0.05
	NIH mice (male, 6/6)	18-20 g	NR	A*β*_1-42_-induced AD model	Sodium pentobarbital	Defatted decoction (0.02 g g^−1^, ig); after the first MWM test; once daily for 3 weeks	Normal saline (same volume, ig) after the first MWM test; once daily for 3 weeks	(1) MWM test (escape latency)	(1) *P* < 0.05
								(2) Beta-amyloid IOD	(2) *P* < 0.05
	NIH mice (male, 6/6)	18-20 g	NR	A*β*_1-42_-induced AD model	Sodium pentobarbital	Essential oil (0.02 g g^−1^, ig); after the first MWM test; once daily for 3 weeks	Normal saline (same volume, ig) after the first MWM test; once daily for 3 weeks	(1) MWM test (escape latency)	(1) *P* < 0.05
								(2) Beta-amyloid IOD	(2) *P* < 0.05

Tian et al. [29]	NIH mice (male, 6/6)	18-20 g	NR	A*β*_1-42_-induced AD model	Sodium pentobarbital	Water extract (0.02 g g^−1^, ig); after the first MWM test; once daily for 3 weeks	Normal saline (0.2 ml/10 g, ig); after surgery; once daily for 3 weeks	(1) MWM test (number of platform crossing)	(1) *P* < 0.05
								(2) NOS activity	(2) *P* < 0.05
	NIH mice (male, 6/6)	18- 0 g	NR	A*β*_1-42_-induced AD model	Sodium pentobarbital	Defatted decoction (0.02 g g^−1^, ig); after the first MWM test; once daily for 3 weeks	Normal saline (0.2 ml/10 g, ig); after surgery; once daily for 3 weeks	(1) MWM test (number of platform crossing)	(1) *P* > 0.05
								(2) MWM test (time spent in target quadrant)	(2) *P* > 0.05
								(3) NOS activity	(3) *P* < 0.05
	NIH mice (male, 6/6)	18-20 g	NR	A*β*_1-42_-induced AD model	Sodium pentobarbital	Essential oil (0.02 g g^−1^, ig); after the first MWM test; once daily for 3 weeks	Normal saline (0.2 ml/10 g, ig); after surgery; once daily for 3 weeks	(1) MWM test (number of platform crossing)	(1) *P* < 0.05
								(2) NOS activity	(2) *P* < 0.05

Zhou et al. [30]	SD rats (male, 10/10)	250 ± 20 g	NR	Scopolamine-induced AD model	NR	Essential oil (12 g kg^−1^, ig); onset the experiment; once daily for 21 d	NS (same volume, ig); onset the experiment; once daily for 21 d	(1) MWM test (escape latency)	(1) *P* < 0.01
								(2) MWM test (number of platform crossing)	(2) *P* < 0.01
								(3) GFAP - positive cells	(3) *P* < 0.01
								(4) SOD content	(4) *P* < 0.05
								(5) MDA content	(5) *P* < 0.05

Wang GM et al., 2017	Kunming mice (mix, 12/12)	5-6 weeks	NR	Chronic restraint stress-induced cognitive impairments mode	NR	Essential oil (4.5 g kg^−1^, ig), onset the experiment; twice daily for 28 d	NS (same volume, ig); onset the experiment; twice daily for 28 d	(1) MWM test (escape latency)	(1) *P* < 0.01
								(2) MWM test (number of platform crossing)	(2) *P* < 0.01
								(3) Body mass	(3) *P* < 0.05
								(4) Plasma cortisol levels	(4) *P* < 0.01

Hu et al. [32]	Kunming mice (male, 11/11)	18-20 g	NR	Sodium nitrite-induced amnesic model	NR	Essential oil (0.053 g kg^−1^, ig); 21 d before sodium nitrite injection; once daily for 21 d	Tween 80 (same volume, ig); 21 d before sodium nitrite injection; once daily for 21 d	(1) SD test (escape latency)	(1) *P* < 0.05
								(2) SD test (number of errors)	(2) *P* < 0.05
	Kunming mice (male, 11/11)	18-20 g	NR	Sodium nitrite-induced amnesic model	NR	Defatted decoction (5 g kg^−1^, ig); 21 d before sodium nitrite injection; once daily for 21 d	Tween 80 (same volume, ig); 21 d before sodium nitrite injection; once daily for 21 d	(1) SD test (escape latency)	(1) *P* < 0.05
								(2) SD test (number of errors)	(2) *P* < 0.05
	Kunming mice (male, 11/11)	18-20 g	NR	Sodium nitrite-induced amnesic model	NR	*α*-Asarone (0.024 g kg^−1^, ig); 21 d before sodium nitrite injection; once daily for 21 d	Tween 80 (same volume, ig); 21 d before sodium nitrite injection; once daily for 21 d	(1) SD test (escape latency)	(1) *P* < 0.05
								(2) SD test (number of errors)	
	Kunming mice (male, 11/11)	18-20 g	NR	Sodium nitrite-induced amnesic model	NR	*β*-Asarone (0.037 g kg^−1^, ig); 21 d before sodium nitrite injection; once daily for 21 d	Tween 80 (same volume, ig); 21 d before sodium nitrite injection; once daily for 21 d	(1) SD test (escape latency)	(1) *P* < 0.05
								(2) SD test (number of errors)	(2) *P* < 0.01
	Kunming mice (male, 11/11)	18-20 g	NR	Ethanol-induced amnesic model	NR	Essential oil (0.053 g kg^−1^, ig); 21 d before ethanol injection; once daily for 21 d	Tween 80 (same volume, ig); 21 d before ethanol injection; once daily for 21 d	(1) SD test (escape latency)	(1) *P* < 0.05
								(2) SD test (number of errors)	(2) *P* < 0.01
	Kunming mice (male, 11/11)	18-20 g	NR	Ethanol-induced amnesic model	NR	Defatted decoction (5 g kg^−1^, ig); 21d before ethanol injection; once daily for 21 d	Tween 80 (same volume, ig); 21 d before ethanol injection; once daily for 21 d	(1) SD test (escape latency)	(1) *P* > 0.05
								(2) SD test (number of errors)	(2) *P* < 0.05
	Kunming mice (male, 11/11)	18-20 g	NR	Ethanol-induced amnesic model	NR	*α*-Asarone (0.024 g kg^−1^, ig); 21 d before ethanol injection; once daily for 21 d	Tween 80 (same volume, ig); 21 d before ethanol injection; once daily for 21 d	(1) SD test (escape latency)	(1) *P* < 0.01
								(2) SD test (number of errors)	
	Kunming mice (male, 11/11)	18-20 g	NR	Ethanol-induced amnesic model	NR	*β*-Asarone (0.037 g kg^−1^, ig); 21 d before ethanol injection; once daily for 21 d	Tween 80 (same volume, ig); 21 d before ethanol injection; once daily for 21 d	(1) SD test (escape latency)	(1) *P* < 0.05
								(2) SD test (number of errors)	(2) *P* < 0.01
	Kunming mice (male, 11/11)	18-20 g	NR	Sodium pentobarbital-induced amnesic model	NR	Essential oil (0.053 g kg^−1^, ig); 21 d before ethanol injection; once daily for 21 d	Tween 80 (same volume, ig); 21 d before ethanol injection; once daily for 21 d	(1) EY-M test (number of errors)	(1) *P* < 0.05
	Kunming mice (male, 11/11)	18-20 g	NR	Sodium pentobarbital-induced amnesic model	NR	Defatted decoction (5 g kg^−1^, ig); 21 d before ethanol injection; once daily for 21 d	Tween 80 (same volume, ig); 21 d before ethanol injection; once daily for 21 d	(1) EY-M test (number of errors)	(1) *P* < 0.05
	Kunming mice (male, 11/11)	18-20 g	NR	Sodium pentobarbital-induced amnesic model	NR	*α*-Asarone (0.024 g kg^−1^, ig); 21 d before ethanol injection; once daily for 21 d	Tween 80 (same volume, ig); 21 d before ethanol injection; once daily for 21 d	(1) EY-M test (number of errors)	(1) *P* < 0.05
	Kunming mice (male, 11/11)	18-20 g	NR	Sodium pentobarbital-induced amnesic model	NR	*β*-Asarone (0.037 g kg^−1^, ig); 21 d before ethanol injection; once daily for 21 d	Tween 80 (same volume, ig); 21 d before ethanol injection; once daily for 21 d	(1) EY-M test (number of errors)	(1) *P* < 0.05

Chen et al. [33]	ICR mice (male, 10/10)	18 ± 1 g	Random number table	D-gal-induced dementia model	NR	Water extract (70, 35, 17.5, or 8.75 mg kg^−1^, ig); 1 week after D-galactose injection; once daily for 7 weeks	Distilled water (same volume, ig); 1 week after D-galactose injection; once daily for 7 weeks	(1) MWM test (escape latency)	(1) *P* > 0.05
								(2) MWM test (number of platform crossing)	(2) *P* > 0.05
								(3) MDA levels	(3) *P* > 0.05
								(4) SOD activity	(4) *P* > 0.05

Gu et al. [34]	ICR mice (male, 10/10)	19.6 ± 1.5 g	Random number table	Scopolamine-induced dysmnesia model	NR	Water extract (70, 35, 17.5, or 8.75 mg kg^−1^, ig); onset the experiment; once daily for 2 weeks	NS (same volume, ig); onset the experiment; once daily for 2 weeks	(1) SD test (escape latency)	(1) *P* < 0.01
								(2) SD test (number of errors)	(2) *P* < 0.01
	ICR mice (male, 10/10)	19.6 ± 1.5 g	Random number table	NaNO_2_-induced dysmnesia model	NR	Water extract (70, 35, 17.5, or 8.75 mg kg^−1^, ig); onset the experiment; once daily for 2 weeks	NS (same volume, ig); onset the experiment; once daily for 2 weeks	(1) SD test (escape latency)	(1) *P* < 0.01
								(2) SD test (number of errors)	(2) *P* < 0.05
	ICR mice (male, 10/10)	19.6 ± 1.5 g	Random number table	Ethanol-induced dysmnesia model	NR	Water extract (70, 35, 17.5, or 8.75 mg kg^−1^, ig); onset the experiment; once daily for 2 weeks	NS (same volume, ig); onset the experiment; once daily for 2 weeks	(1) ST test (escape latency)	(1) *P* < 0.01
								(2) ST test (number of errors)	(2) *P* < 0.01
								(3) AchE activity	(3) *P* > 0.05
	Wistar rats (male, 10/10)	200 ± 25 g	NR	Scopolamine-induced dysmnesia model	NR	Water extract (35, 17.5, or 8.75 mg kg^−1^, ig); onset the experiment; once daily for 4 weeks	NS (same volume, ig); onset the experiment; once daily for 2 weeks	(1) MWM test (escape latency)	(1) *P* < 0.01
								(2) MWM test (number of platform crossing)	(2) *P* < 0.01

Wu et al., 2004	Aged NIH mice (male, 10/10)	NR	NR	Aged mice	NR	Essential oil (0. 01075 ml g^−1^, ig); onset the experiment; twice daily for 10 d	NS (same volume, ig); onset the experiment; once daily for 10 d	(1) MWM test (escape latency)	(1) *P* < 0.05
								(2) AchE activity	(2) *P* < 0.05
								(3) C-jun express	(3) *P* < 0.05
	Aged NIH mice (male, 10/10)	NR	NR	Aged mice	NR	*β*-Asarone (0. 01075 ml g^−1^, ig); onset the experiment; twice daily for 10 d	NS (same volume, ig); onset the experiment; once daily for 10 d	(1) MWM test (escape latency)	(1) *P* > 0.05
								(2) MWM test (number of errors)	(2) *P* > 0.05
								(3) AchE activity	(3) *P* > 0.05
								(4) C-jun express	(4) *P* > 0.05
	Aged NIH mice (male, 10/10)	NR	NR	Aged mice	NR	Water extract (0. 01075 ml g^−1^ g, ig); onset the experiment; twice daily for 10 d	NS (same volume, ig); onset the experiment; once daily for 10 d	(1) MWM test (escape latency)	(1) *P* > 0.05
								(2) AchE activity	(2) *P* > 0.05
								(3) C-jun express	(3) *P* < 0.05
	Kunming mice (male, 10/10)	NR	NR	Ethanol-induced dysmnesia model	NR	Water extract (0. 01075 ml g^−1^, ig); onset the experiment; twice daily for 10 d	NS (same volume, ig); onset the experiment; once daily for 10 d	(1) ST test (escape latency)	(1) *P* < 0.05
	Kunming mice (male, 10/10)	NR	NR	NaNO_2_-induced dysmnesia model	NR	Essential oil (0. 01075 ml g^−1^, ig); onset the experiment; twice daily for 10 d	NS (same volume, ig); onset the experiment; once daily for 10 d	(1) SD test (escape latency)	(1) *P* > 0.05
								(2) SD test(number of errors)	(2) *P* > 0.05
	Kunming mice (male, 10/10)	NR	NR	NaNO_2_-induced dysmnesia model	NR	*β*-Asarone (0. 01075 ml g^−1^, ig); onset the experiment; twice daily for 10 d	NS (same volume, ig); onset the experiment; once daily for 10 d	(1) SD test (escape latency)	(1) *P* < 0.05
								(2) SD test (number of errors)	(2) *P* > 0.05
	Kunming mice (male, 10/10)	NR	NR	NaNO_2_-induced dysmnesia model	NR	Water extract (0. 01075 ml g^−1^, ig); onset the experiment; twice daily for 10 d	NS (same volume, ig); onset the experiment; once daily for 10 d	(1) SD test (escape latency)	(1) *P* < 0.05
								(2) SD test (number of errors)	(2) *P* > 0.05
	Kunming mice (male, 10/10)	NR	NR	Scopolamine-induced dysmnesia model	NR	Essential oil (0. 01075 ml g^−1^, ig); onset the experiment; twice daily for 10 d	NS (same volume, ig); onset the experiment; once daily for 10 d	(1) SD test (escape latency)	(1) *P* < 0.05
								(2) SD test (number of errors)	(2) *P* > 0.05
	Kunming mice (male, 10/10)	NR	NR	Scopolamine-induced dysmnesia model	NR	*β*-Asarone (0. 01075 ml g^−1^, ig); onset the experiment; twice daily for 10 d	NS (same volume, ig); onset the experiment; once daily for 10 d	(1) SD test (escape latency)	(1) *P* < 0.05
								(2) SD test (number of errors)	(2) *P* > 0.05
	Kunming mice (male, 10/10)	NR	NR	Scopolamine-induced dysmnesia model	NR	Water extract (0. 01075 ml g^−1^, ig); onset the experiment; twice daily for 10 d	NS (same volume, ig); onset the experiment; once daily for 10 d	(1) SD test (escape latency)	(1) *P* < 0.05
								(2) SD test(number of errors)	(2) *P* > 0.05

Wen et al., 2009	ICR mice (mix, 10/10)	20 ± 2 g	NR	Ethanol-induced dysmnesia model	NR	Water extract (3 and 12 g kg^−1^, ig); onset the experiment; twice daily for 14 d	NS (same volume, ig); onset the experiment; twice daily for 14 d	(1) ST test (escape latency)	(1) *P* < 0.01
	ICR mice (mix, 10/10)	20 ± 2 g	NR	NaNO_2_-induced dysmnesia model	NR	Essential oil (3 and 12 g kg^−1^, ig); onset the experiment; once daily for 14 d	NS (same volume, ig); onset the experiment; twice daily for 14 d	(2) EY-M test (number of errors)	(1) *P* < 0.05
	ICR mice (mix, 10/10)	20 ± 2 g	NR	Scopolamine-induced dysmnesia model	NR	Water extract (3 and 12 g kg-^1^, ig); onset the experiment; once daily for 14 d	NS (same volume, ig); onset the experiment; twice daily for 14 d	(1) SD test (escape latency)	(1) *P* < 0.05
								(2) SD test (number of errors)	(2) *P* < 0.05
	ICR mice (mix, 10/10)	20 ± 2 g	NR	Scopolamine-induced dysmnesia model	NR	Essential oil (3 and 12 g kg^−1^, ig); onset the experiment; once daily for 14 d	NS (same volume, ig); onset the experiment; twice daily for 14 d	(1) SD test (escape latency)	(1) *P* < 0.05
								(2) SD test (number of errors)	(2) *P* < 0.01
	ICR mice (mix, 10/10)	20 ± 2 g	NR	Scopolamine-induced dysmnesia model	NR	Water extract (3 and 12 g kg^−1^, ig); onset the experiment; once daily for 14 d	NS (same volume, ig); onset the experiment; twice daily for 14 d	(1) MWM test (escape latency)	(1) *P* < 0.01
	ICR mice (mix, 10/10)	20 ± 2 g	NR	Scopolamine-induced dysmnesia model	NR	Essential oil (3 and 12 g kg^-1,^ ig); onset the experiment; once daily for 14 d	NS (same volume, ig); onset the experiment; twice daily for 14 d	(1) MWM test (escape latency)	(1) *P* < 0.01

Yang et al. [37]	SD rats (male, 12/12)	250 ± 30 g	NR	A*β*_1-42_-induced AD model	NR	*β*-Asarone (10, 20, and 30 mg kg^−1^, ig); after the model finished; twice daily for 28 d	NS (same volume, ig); after the model finished; once daily for 28 d	(1) MWM test (escape latency)	(1) *P* < 0.05
								(2) MWM test (number of platform crossing)	(2) *P* < 0.01
								(3) Astrocyte activity	(3) *P* < 0.01

Zhou et al. [38]	SD rats (male,10/10)	200-250 g	NR	D-gal- and AlCl_3_-induced AD model	NR	*α*-Asarone (10, 25 mg kg^−1^, ip); after the after model finished; once daily for 28 d	NS (same volume, ip); after the model finished; once daily for 28 d	(1) MWM test (number of platform crossing)	(1) *P* < 0.01
								(2) A*β* and tau protein expression	(2) *P* < 0.01
								(3) ACh levels	(3) *P* < 0.05
								(4) AChE levels	(4) *P* > 0.05
								(5) ChAT levels	(5) *P* > 0.05

Jiang et al., 2007	Kunming mice (mix, 10/10)	18-20 g	NR	AlCl_3_-induced AD model	NR	*β*-Asarone (1.06, 2.12, and 4.24 mg 100 g^−1^, ig); after the model finished; once daily for 2 months	NS (same volume, ig); after the model finished; once daily for 2 months	(1) MWM test (number of errors)	(1) *P* < 0.01
								(2) SOD levels	(2) *P* < 0.01
								(3) MAD levels	(3) *P* < 0.01

Huang et al. [40]	FMR1gene knock mice (16/17)	17-18 g	NR	Fragile X syndrome model	NR	*α*-Asarone (3, 6, 9, 12, 24 mg kg^−1^, ip); onset the experiment; once daily for 8 d	NS (same volume, ip); onset the experiment; once daily for 8 d	(1) SD test (number of errors)	(1) *P* > 0.05
								(2) P-Akt expression	(2) *P* < 0.05
								(3) Akt expression	(3) *P* > 0.05

Wang BL et al., 2017	SD rats (male, 15/15)	280 ± 20 g	Random number table	A*β*_1-42_-induced AD model	Phenytoin sodium	*β*-Asarone (10, 20, and 30 mg kg^−1^, ig); after the model finished; once daily for 4 weeks	NS (same volume, ig); after the model finished; once daily for 4 weeks	(1) MWM test (escape latency)	(1) *P* < 0.01
								(2) MWM test (number of platform crossing)	(2) *P* < 0.01
								(3) HIF levels	(3) *P* < 0.05

Guo et al. [42].	Kunming mice (male, 11/11)	25 ± 5 g	Random block allocation method	Scopolamine-induced AD model	NR	*β*-Asarone (21.2 mg kg^−1^, ig); after the model finished; once daily for 14 d	NS (same volume, ig); after the model finished; once daily for 14 d	(1) MWM test (escape latency)	(1) *P* < 0.01

Jiang et al. [43]	Wistar rats (mix, 8/8)	250-300 g	NR	STZ-induced AD model	NR	Essential oil (5, 10 and 20 g kg-^1^, ig); onset the experiment; once daily for 20 d	Solvent (same volume, ig); onset the experiment; once daily for 20 d	(1) MWM test (escape latency)	(1) *P* < 0.01
								(2) SOD levels	(2) *P* < 0.01
								(3) MAD levels	(3) *P* < 0.01

Yang et al. [44]	Wistar rats (10/10)	35 ± 5 g	NR	PTZ-induced epilepsy model	NR	*α*-Asarone (29 mg kg^−1^, ig); after PTZ injection; twice daily for 7 d	NS (same volume, ig); after PTZ injection; twice daily for 7 d	(1) MWM test (number of platform crossing)	(1) *P* < 0.05
								(2) MWM test (time spent in target quadrant)	(2) *P* < 0.05
	Wistar rats (10/10)	35 ± 5 g	NR	PTZ-induced epilepsy model	NR	AGA (2.35 g kg^−1^, ig); after PTZ injection; twice daily for 7 d	NS (same volume, ig); after PTZ injection; twice daily for 7 d	(1) MWM test (number of platform crossing)	(1) *P* < 0.05
								(2) MWM test (time spent in target quadrant)	(2) *P* < 0.05

Wang et al. [45]	ICR mice (mix, 10/10)	20 ± 2 g	NR	Scopolamine-induced dysmnesia model	NR	Essential oil (100, 150, and 300 mg kg-^1^, ig); before the experiment; once daily for 7 d	NS (same volume, ig); before the experiment; once daily for 7 d	(1) MWM test (escape latency)	(1) *P* < 0.01
								(2) MWM test (number of platform crossing)	(2) *P* < 0.01
								(3) MWM test (time spent in target quadrant)	(3) *P* < 0.01

Ma et al. [46]	SD rats (male, 6/6)	260-280 g	NR	A*β*_1-42_-induced AD model	Sodium pentobarbital	*β*-Asarone (12.5, 25, 50 mg kg^−1^, ig); after the model finished; once daily for 4 weeks	NS (same volume, ig); after the model finished; once daily for 4 weeks	(1) MWM test (escape latency)	(1) *P* < 0.05
								(2) GA P-43 mRNA levels	(2) *P* < 0.05
								(3) SYP mRNA levels	(3) *P* < 0.05
								(4) PSD-95 mRNA levels	

Ach: acetylcholine; AchE: acetylcholinesterase; SD rats: Sprague-Dawley rats; NIH mice: National Institutes of Health mice; SAMP8 mice: senescence-accelerated mouseprone 8 mice; AD: Alzheimer's disease; AlCl_3_: aluminum trichloride; ChAT: acetylcholine transferase; D-gal: D-galactose; i.g.: intragastrical injection; i.p.: intraperitoneal injection; i.h.: hypodermic injection; MWM test: Morris water maze test; MCAO: middle cerebral artery occlusion; MDA: malondialdehyde; GSH-PX: glutathione peroxidase; NR: not report; SD test: step down test; STZ: streptozotocin; SOD: superoxide dismute; HIF: hypoxia-inducible factor; GSH-Px: glutathione peroxidase; NE: norepinephrine; 5-TH: 5-hydroxytryptamine; DA: dopamine; SYN/SYN: synaptophysin; NOS: nitric oxide synthase; Bcl-2: B-cell lymphoma/leukemia-2; MAP2: microtubule-associated protein 2; RAM: radial eight-arm maze; EY-M: electric Y-maze; A*β*1-42: amyloid beta 1-42; PTZ: pent ylenetet razol; NS: normal saline.

**Table 2:** Quality assessment of included studies.

Study (years)	1	2	3	4	5	6	7	8	9	10	Total
Yang et al. [17]	**√**	**√**	**√**			**√**			**√**		5
Wei et al., 2013	**√**	**√**	**√**								3
Sundaramahalingam et al. [18]	**√**	**√**							**√**		3
Shin et al. [19]	**√**	**√**	**√**						**√**		4
Ma et al. [11]	**√**		**√**			**√**			**√**	**√**	5
Liu et al. [20]	**√**	**√**	**√**			**√**			**√**	**√**	6
Limón et al. [21]	**√**	**√**	**√**			**√**			**√**		5
Li et al. [22]	**√**	**√**	**√**			**√**			**√**		5
Zhang et al. [5]	**√**		**√**						**√**		3
Lee et al. [10]	**√**	**√**				**√**			**√**		4
Lee et al. [23]	**√**	**√**	**√**		**√**	**√**					5
Kumar et al., 2012	**√**	**√**							**√**		3
Kim et al. [25]	**√**	**√**				**√**					3
Geng et al. [26]	**√**	**√**	**√**			**√**					4
Chen et al. [27]	**√**		**√**						**√**		3
Ma et al. [28]	**√**		**√**			**√**			**√**	**√**	5
Tian et al. [29]	**√**					**√**					2
Zhou et al. [30]	**√**	**√**	**√**								3
Wang GM et al., 2017	**√**		**√**								2
Hu et al. [32]	**√**		**√**								2
Chen et al. [33]	**√**		**√**								2
Gu et al. [34]	**√**		**√**								2
Wu et al., 2004	**√**		**√**								2
Wen et al., 2009	**√**		**√**								2
Yang et al. [37]	**√**	**√**	**√**		**√**				**√**	**√**	6
Zhou et al. [38]	**√**		**√**								2
Jiang et al., 2007	**√**		**√**								2
Huang et al. [40]	**√**										1
Wang BL et al., 2017	**√**	**√**	**√**			**√**			**√**		5
Guo et al. [42]	**√**		**√**								2
Jiang et al. [43]	**√**		**√**						**√**		3
Yang et al. [44]	**√**		**√**								2
Wang et al. [45]	**√**	**√**	**√**								3
Ma et al. [46]	**√**		**√**			**√**			**√**		4

1: peer-reviewed publication; 2: statements describing control of temperature; 3: randomization to treatment group; 4: allocation concealment; 5: blinded assessment of outcome; 6: avoidance of anesthetics with known notable intrinsic neuroprotective properties; 7: use of animals with relevant comorbidities; 8: sample size calculation; 9: compliance with animal welfare regulations; 10: declared any potential conflict of interest.

**Table 3:** Characteristics of mechanism studies of EAAGA on cognition impairment.

Study (years)	Model	Method of administration (experimental group versus control group)	Observations	Possible mechanisms
Yang et al. [17]	Chronic lead-induced dysmnesia model	*β*-Asarone versus distilled water	Attenuated memory deficits	Arc/Arg3.1 and Wnt pathway
			Increased dendritic spine density	Increased dendritic spine density
			Up-regulated NR2B, Arc/Arg3.1, and Wnt7a protein expression	

Wei et al., 2013	A*β*PP/PS1 double-transgenic mice	*β*-Asarone versus Tween 80	Improved cognitive function	CaMKII/CREB/Bcl-2 signaling pathway
			Prevents PC12 cell and cortical neuron damage	Inhibition of apoptosis
			Inhibited the apoptosis of PC12 cells and cortical neurons	

Sundaramahalingam et al. [18]	Noise stress induced memory impairment model	*α*-Asarone versus Tween 80	Prevent memory impairment	Reduction of oxidative reactions
			Decreased hsp 70 mRNA levels	
			Decreased SOD and AChE activity	
			Increased CAT and G6PD activity	
			Increased VC, VE, and GSH levels	

Shin et al. [19]	LPS-induced cognitive handicap mode	*α*-Asarone versus NS	Ameliorated memory deficits	Repression of inflammatory reactions
			Reduced Iba1 protein expression	Inhibition of apoptosis
			Reduced TNF-*α* and IL-1*β* mRNA	
			Reduced BACE1 expression	
			Increased CA1 neurons	
			Reduced TUNEL-labeled cells	

Ma et al. [11]	A*β*_1-42_-induced AD model	Water extract versus NS	Ameliorated memory deficits	Inhibition of neurotoxicity
		Essential oil versus NS	Reduced A*β* positive cells	
		Defatted decoction versus NS	Decreased DCx and nestin expression	
			Decreased nestin positive cells	

Liu et al. [20]	APPswe/PS1dE9 double transgenic mice	*β*-Asarone versus Tween 80	Improved the learning and memory ability	Regulation of synaptic plasticity
			Increased SYP and GluR1 expression	

Limón et al. [21]	A*β*-induced AD model	*α*-Asarone versus NS	Ameliorated memory deficits	Reduction of oxidative reactions
			Decreased NO levels	

Li et al. [22]	D-gal- and AlCl3-induced AD model	*β*-Asarone versus NS	Improved the learning and memory ability	Protection of cerebrovascular
			Increased rCBF and the activity of Na–K-ATP	
			Decreased pyruvic acid contents	
			Decreased ET-1, eNOS, and APP mRNA expression	

Zhang et al. [5]	Aged mice	Essential oil versus Tween 80	Improved cognitive function Increased 5-HT, NE, DA, and NE levels Decreased AChE activity	Improvement of cognitive function
	Scopolamine-induced dysmnesia model	Essential oil versus Tween 80		
	Ethanol-induced dysmnesia model	Essential oil versus Tween 80		
	Aged rats	Essential oil versus Tween 80		
	Sodium nitrite-induced dysmnesia model	Essential oil versus Tween 80		

Lee et al. [10]	MCAO/2 h-induced cognitive impairments model	AGA versus NS	Attenuated learning and memory deficits Increased cell density	Inhibition of apoptosis

Lee et al. [23]	Chronic corticosterone exposed	*β*-Asarone versus NS	Improved cognitive function Increased BDNF and CREB expression Increased BDNF, CREB, and Bcl-2 mRNAs levels Decreased Bax mRNAs levels Decreased serum levels of CORT	Inhibition of apoptosis

Kumar et al., 2012	Scopolamine-induced amnesic model	*α*-Asarone versus vehicle	Improved cognitive function Increased of AchE activity Inhibition MDA expression and SOD levels Reduced SOD activity	Reduction of oxidative reactions

Kim et al. [25]	Ibotenic acid-induced amnesia	AGA versus NS	Ameliorated learning and memory deficits Increased ChAT positive neurons Increased AchE neurons	Stimulation of cholinergic system

Geng et al. [26]	A*β*_1-42_-induced AD rat model	*β*-Asarone versus NS	Ameliorated learning and memory deficits Increased Bcl-2, Bcl-w expression Increased Bcl-2 and Bcl-w mRNA levels Decreased cleavage of caspase-3 Reduced caspase-3 mRNA levels Decreased p-JNK expression	Inhibition of apoptosis

Chen et al. [27]	SAMP8 mice	*β*-Asarone versus NS	Improved cognitive function Reduced ROCK, beclin1, and LC3 expression Increased p62 expression Increased GAP43, MAP2, and SYN expression Increased GAP43-, MAP2-, and SYN-positive cells Decreased lipofuscin-positive cells	Reduction of autophagy

Ma et al. [28]	A*β*-induced AD model	Water extract versus NS	Ameliorated learning and memory deficits Decreased A*β* plaques depositions	Improvement of cognitive function
		Water extract without oil versus NS		
		Essential oil versus NS		

Tian et al. [29]	A*β*-induced AD model	Water extract versus NS	Ameliorated learning and memory deficits Decreased NOS activity	Inhibition of neurotoxicity
		Defatted decoction versus NS		
		Essential oil versus NS		

Zhou et al. [30]	Scopola-induced AD model	Essential oil versus NS	Ameliorated learning and memory deficits Decreased GFAP expression Decreased MDA levels Increased SOD levels	Reduction of oxidative reactions

Wang GM et al., 2017	Chronic restraint stress-induced cognitive impairments mode	Essential oil versus NS	Ameliorated learning and memory deficits Increased body mass Decreased plasma cortisol levels	Inhibition of chronic stress

Hu et al. [32]	Sodium nitrite-induced amnesic model	Essential oil versus Tween 80	Increased learning and memory deficits	Improvement of cognitive function
		Defatted decoction versus Tween 80		
		*α*-Asarone versus Tween 80		
		*β*-Asarone versus Tween 80		
	Ethanol-induced amnesic model	Essential oil versus Tween 80	Ameliorated learning and memory deficits	Improvement of cognitive function
		Defatted decoction versus Tween 80		
		*α*-Asarone versus Tween 80		
		*β*-Asarone versus Tween 80		

Chen et al. [33]	D-galactose-induced dementia model	Water extract versus distilled water	Ameliorated memory deficits Decreased MDA levels Increased SOD activity	Reduction of oxidative reactions

Gu et al. [34]	Scopolamine-induced dysmnesia mice	Water extract versus NS	Ameliorated memory deficits	Improvement of cognitive function
	NaNO_2_-induced dysmnesia model	Water extract versus NS	Ameliorated memory deficits	
	45% ethanol-induced dysmnesia mice	Water extract versus NS	Ameliorated memory deficits The AchE activity of mice brain was not influenced	
	Scopolamine-induced dysmnesia rat	Water extract versus NS	Ameliorated memory deficits	

Wu et al., 2004	Aged mice	Essential oil versus NS	Ameliorated memory deficits Decreased AChE activity Increased c-jun mRNA levels	Inhibition of apoptosis
	Aged mice	*β*-Asarone versus NS		
	Aged mice	Water extract versus NS		
	Ethanol-induced dysmnesia model	Water extract versus NS		
	NaNO_2_-induced dysmnesia model	Essential oil versus NS		
	NaNO_2_-induced dysmnesia model	*β*-Asarone versus NS		
	NaNO_2_-induced dysmnesia model	Water extract versus NS	Ameliorated memory deficits	
	Scopolamine-induced dysmnesia model	Essential oil versus NS		
	Scopolamine-induced dysmnesia model	Essential oil versus NS		
	Scopolamine-induced dysmnesia model	Water extract versus NS		

Wen et al., 2009	Ethanol-induced dysmnesia model	Water extract versus NS	Ameliorated memory deficits	Inhibition of apoptosis
	NaNO_2_-induced dysmnesia model	Essential oil versus NS		
	Scopolamine-induced dysmnesia model	Water extract versus NS		
	Scopolamine-induced dysmnesia model	Essential oil versus NS		
	Scopolamine-induced dysmnesia model	Water extract versus NS		
	Scopolamine-induced dysmnesia model	Essential oil versus NS		

Yang et al. [37]	A*β*_1-42_-induced AD model	*β*-Asarone versus NS	Improved cognitive function Inhibited AQP4, IL-1*β*, and TNF-*α* expression Decreased A*β* deposition Alleviated hippocampal damage	Suppression of astrocyte activation

Zhou et al. [38]	D-gal- and AlCl3-induced AD model	*α*-Asarone versus NS	Improved cognitive function Decreased A*β* and Tau protein expression Increased ACh expression	Inhibition of apoptosis

Jiang et al. 2007	AlCl_3_-induced AD model	*β*-Asarone versus NS	Improved cognitive function Increased SOD and GSH-Px levels Decreased MAD levels	Reduction of oxidative reactions

Huang et al. [40]	Fragile X syndrome model	*α*-Asarone versus NS	Improved cognitive function	Damage of Akt pathway

Wang BL et al., 2017	A*β*_1-42_-induced AD model	*β*-Asarone versus NS	Improved cognitive function Decreased HIF and MDA levels Increased SOD and CAT levels	Reduction of oxidative reactions

Guo et al. [42]	Scopolamine-induced AD model	*β*-Asarone versus NS	Improved cognitive function	Inhibition of apoptosis

Jiang et al. [43]	STZ-induced AD model	Essential oil versus solvent	Improved cognitive function Decreased MDA levels Increased SOD levels	Reduction of oxidative reactions

Yang et al. [44]	PTZ-induced epilepsy model	AGA versus NS	Improved cognitive function	Inhibition of apoptosis
	PTZ-induced epilepsy model	*α*-Asarone versus NS	Improved cognitive function	Inhibition of apoptosis

Wang et al. [45]	A*β*_1-42_-induced AD model	Essential oil versus NS	Improved cognitive function	Improvement of cognitive function

Ma et al. [46]	D-gal- and AlCl_3_-induced AD model	*β*-Asarone versus NS	Improved cognitive function Decreased GA P-43 mRNA levels Increased SYP mRNA levels Decreased PSD-95 mRNA levels	Regulation of synaptic plasticity

Ach: acetylcholine; AchE: acetylcholinesterase; AD: Alzheimer's disease; AlCl_3_: aluminum trichloride; ChAT: acetylcholine transferase; D-gal: D-galactose; MCAO: middle cerebral artery occlusion; MDA: malondialdehyde; STZ: streptozotocin; SOD: superoxide dismute; HIF: hypoxia-inducible factor; SYN/SYN: synaptophysin; MAP2: microtubule-associated protein 2; A*β*1-42: amyloid beta 1-42; NS: normal saline; PTZ: pent ylenetet razol; GSH-Px: glutathione peroxidase; NE: norepinephrine; 5-TH: 5-hydroxytryptamine; DA: dopamine; NOS: nitric oxide synthase; Bcl-2: B-cell lymphoma/leukemia-2.
